# Pre-exposure prophylaxis (PrEP) for HIV prevention among people who inject drugs: a global mapping of service delivery

**DOI:** 10.1186/s12954-023-00729-6

**Published:** 2023-02-13

**Authors:** Graham Shaw, Robin Schaefer, Heather-Marie A Schmidt, Annie Madden, Judy Chang, Antons Mozalevskis, Busisiwe Msimanga-Radebe, Nabeel Mangadan Konath, Annette Verster, Rachel Baggaley, Michelle Rodolph, Virginia Macdonald

**Affiliations:** 1 Independent Consultant, Cambridge, UK; 2 Global HIV, Hepatitis and STIs Programmes, World Health Organization, Avenue Appia 20, 1211 Geneva, Switzerland; 3 UNAIDS Regional Office for Asia and the Pacific, Bangkok, Thailand; 4 International Network of People who Use Drugs, London, UK; 5 World Health Organization, Pretoria, South Africa; 6 World Health Organization, Yangon, Myanmar

**Keywords:** HIV prevention, Pre-exposure prophylaxis, People who inject drugs, Harm reduction

## Abstract

**Background:**

The World Health Organization (WHO) recommends oral pre-exposure prophylaxis (PrEP) for all people at substantial risk of HIV as part of combination prevention. The extent to which this recommendation has been implemented globally for people who inject drugs is unclear. This study mapped global service delivery of PrEP for people who inject drugs.

**Methods:**

Between October and December 2021, a desk review was conducted to obtain information on PrEP services for people who inject drugs from drug user-led networks and HIV, harm reduction, and human rights stakeholders. Websites of organizations involved in HIV prevention or services for people who inject drugs were searched. Models of service delivery were described in terms of service location, provider, and package.

**Results:**

PrEP services were identified in 27 countries (15 high-income). PrEP delivery models varied within and across countries. In most services, PrEP services were implemented in healthcare clinics without direct links to other harm reduction services. In three countries, PrEP services were also provided at methadone clinics. In 14 countries, PrEP services were provided through community-based models (outside of clinic settings) that commonly involved peer-led outreach activities and integration with harm reduction services.

**Conclusions:**

This study indicates limited PrEP availability for people who inject drugs. There is potential to expand PrEP services for people who inject drugs within harm reduction programs, notably through community-based and peer-led services. PrEP should never be offered instead of evidence-based harm reduction programs for people who inject drugs; however, it could be offered as an additional HIV prevention choice as part of a comprehensive harm reduction program.

**Supplementary Information:**

The online version contains supplementary material available at 10.1186/s12954-023-00729-6.

## Introduction

People who inject drugs are at 35 times higher risk of HIV acquisition than the general population [1], primarily due to sharing injecting equipment, largely driven by poor access to sterile injecting equipment by people who inject drugs [2, 3], as well as sexual behaviors [4, 5]. These risks are driven by underlying structural factors, including stigma and discrimination, criminalization [6], incarceration [7], and violence [8, 9].

Since 2015, the World Health Organization (WHO) has recommended offering tenofovir disoproxil fumarate (TDF)-based oral pre-exposure prophylaxis (PrEP) to all people at substantial risk of HIV as part of combination HIV prevention. Although there is strong evidence that oral PrEP is highly effective at preventing sexual HIV acquisition [10], evidence is scarce for the prevention of parenteral HIV acquisition. However, pharmacokinetic/pharmacodynamic modeling suggests that oral PrEP could provide high levels of protection against HIV infection from injecting behaviors [11]. One randomized controlled trial of oral PrEP among people who inject drugs in Thailand reported a reduction in HIV infections [12], but the study had severe limitations in providing insight into the efficacy of oral PrEP at preventing parenteral infection [13].

There are significant structural impediments faced by people who inject drugs, particularly the criminalization of drug possession and use and human rights violations severely limiting their access to essential, evidence-based harm reduction services. These services include needle and syringe programs (NSPs) and opioid agonist maintenance therapy (OAMT), and other health services, such as community distribution of naloxone to manage opioid overdose, services for hepatitis C virus (HCV) (testing, diagnosis, and treatment), HIV testing and counseling as well as antiretroviral therapy (ART) for people who inject drugs who are living with HIV [2, 3]. The WHO consolidated guidelines on HIV, viral hepatitis and sexually transmitted infection (STI) prevention, diagnosis, treatment, and care for key populations includes PrEP in the recommended package of interventions for people who inject drugs as an additional prevention choice that can complement comprehensive harm reduction services [14]; however, the guidelines emphasize that qualitative research found that people who inject drugs prioritize access to evidence-based harm reduction services over PrEP. Therefore, although PrEP can be framed as complementary to the provision of comprehensive harm reduction interventions, it should never be positioned or provided as an alternative to harm reduction approaches among people who inject drugs [15].

Even when made available, people who inject drugs may face considerable barriers to PrEP access and uptake, including anticipated or experienced stigma and discrimination by healthcare providers [16–18]. A systematic review of studies in the USA found that people who inject drugs expressed high willingness to use PrEP but very low actual uptake [19]. Several studies found that awareness of and willingness to use PrEP by people who inject drugs was higher when this was discussed in NSPs or where PrEP was provided at NSPs [20–24], which are commonly provided within a non-judgmental framework and so address structural barriers to accessing health services such as stigma and discrimination. This suggests that harm reduction may represent an important opportunity for integration with PrEP to address the complex health needs of people who inject drugs. However, few studies considered willingness to use PrEP outside of the USA [25, 26], and little is known about the extent to which the WHO PrEP recommendations have been implemented for people who inject drugs worldwide. To inform global implementation guidance for PrEP by WHO and determine whether additional efforts are needed to promote PrEP as an HIV prevention option in addition to existing harm reduction interventions for people who inject drugs, we conducted a desk review to map global service delivery of PrEP for this population.

## Methods

Between October and December 2021, information on the delivery of PrEP services for people who inject drugs was requested from drug user-led networks and groups and other stakeholders and networks in the sectors of HIV, sexual and reproductive health and rights, harm reduction, and human rights. Additional file 1 lists all groups and organizations contacted. In addition, a search of relevant information on websites of organizations involved in HIV prevention and PrEP-related studies or services for people who inject drugs was conducted (see Additional file 1 details). All organizations and individuals were contacted with a standardized set of questions that covered key parameters, including service location and service delivery model, populations targeted and number of clients, and funding (see Additional file 1). The collected information was used to qualitatively describe PrEP service delivery models for people who inject drugs in countries where these services were identified according to service location, provider, and package (including integration with harm reduction services).

## Results

**Fig. 1:**
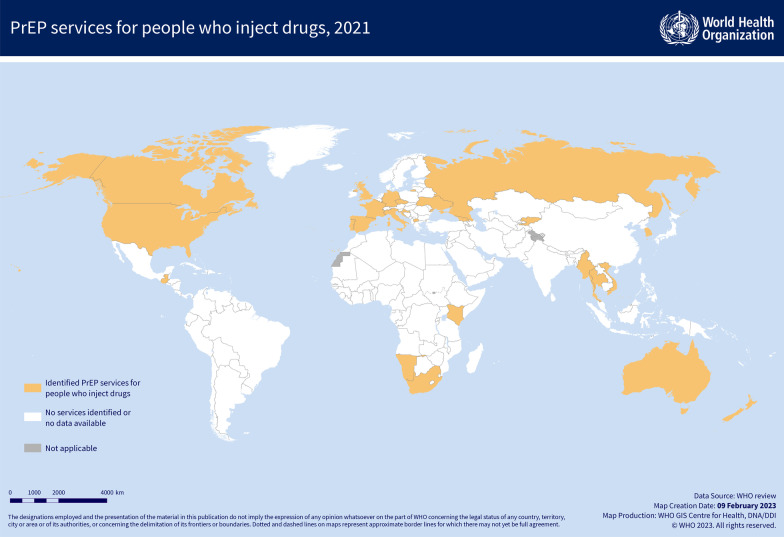
Countries with identified pre-exposure prophylaxis (PrEP) services for HIV prevention for people who inject drugs, 2021. In several identified countries, information relate to services implemented only in specific areas or cities, although whole countries are indicated in the map.

PrEP services for people who inject drugs were identified in 27 countries (15 of which were high-income countries) (Fig. 1). This included a planned PrEP service involving people who inject drugs in Kachin State, Myanmar, that is delayed due to security concerns. In many of the identified countries, these PrEP services were small scale and only available in a limited number of locations (for instance, data obtained for PrEP services for people who inject drugs in the Russian Federation relate only to Saint Petersburg). Numbers of people who inject drugs enrolled in PrEP services were only available for Glasgow, Scotland (47 people who inject drugs had been enrolled between November 2018 and November 2020 [27]), South Africa (by the end of 2020, PrEP was accessed by 6,080 people who inject drugs), Ukraine (70 people who inject drugs had been initiated on PrEP in 2020), and Kyrgyzstan (21 people who inject drugs used PrEP in 2021).

In high-income countries, PrEP services for people who inject drugs were funded through governments or health insurance. In low- and middle-income countries, funding was primarily from external donors. The most common external donor was the US President’s Emergency Plan for AIDS Relief (PEPFAR), which funded services in South Africa, Mozambique, Viet Nam, and Zambia. The Global Fund funded PrEP for people who inject drugs in Georgia, Kenya, and Ukraine.

Data on PrEP service delivery models were available for 24 countries (insufficient data for Georgia, Guatemala, and North Macedonia). Delivery models varied widely across countries regarding service location, service provider, and service package; commonly multiple delivery models were implemented within the one country (Table 1). In nearly all identified countries, PrEP service models for people who inject drugs were delivered in fixed healthcare facilities without direct links to other harm reduction services, involving a medical doctor responsible for PrEP prescribing. In three countries (Kyrgyzstan, Ukraine, and the USA), PrEP services were also delivered at methadone clinics. In nearly all low- and middle-income countries (7/9) and just under half of high-income countries (7/15) with available data, different forms of community-based PrEP delivery models were implemented where services were provided outside of clinic settings (e.g., in community-based drop-in centers), which usually also provided other HIV and harm reduction services. These models commonly involved peer-led outreach activities to reach clients and to link them to services provided at community sites or healthcare clinics (hybrid models). In two countries (Russian Federation [28] and Ukraine), PrEP services for people who inject drugs were provided online, provided either through non-governmental organizations (NGOs) or the private sector (allowing for private purchasing of PrEP), which involved web outreach workers providing people who use drugs with instructions on how to obtain services offline at NGO or affiliated clinics, including for those who wish to access PrEP privately [28].

**Table 1:** Service delivery models for pre-exposure prophylaxis (PrEP) for HIV prevention for people who inject drugs identified in this study

Model	Key characteristics	Countries^[a,b]^	
		High-income	Low- and middle-income
Clinic-based PrEP delivery	• Delivered at fixed healthcare facilities, e.g., hospitals, sexual and reproductive health clinics • Commonly involves specialist HIV or sexual health doctor responsible for PrEP prescribing • Medication dispensed tends to be dispensed at the same healthcare facility or affiliated pharmacies • Usually no other harm reduction services available at the same site	Australia, Croatia, UK (England and Scotland)^[c]^, France, Germany, Italy, Luxembourg, Malta, New Zealand, Portugal, South Korea, USA	Kenya, Namibia, Thailand, Viet Nam
Delivery linked with drug dependence treatment services	• Delivered at fixed healthcare facilities where drug dependence treatment services are provided (methadone clinics)	USA	Kyrgyzstan, Ukraine
Community-based PrEP delivery and peer-led outreach	• PrEP delivered in community-based clinics or drop-in centers and community pharmacies • PrEP services usually linked with other harm reduction services provided in the community settings, especially needle and syringe programs [25] • Peers may be used to increase PrEP awareness through outreach [26, 28] and facilitate linkage to community-based clinics or drop-in centers	Canada, Czech Republic, France, Spain, UK (Scotland)^[c]^, USA	Kenya, Namibia, South Africa, Thailand, Ukraine, Viet Nam
Hybrid model	• Hybrid model where community-based outreach activities, often involving peers, provide linkages to healthcare facilities where PrEP is initiated • PrEP drugs are usually dispensed at a community pharmacy • May involve non-clinical staff to facilitate PrEP initiation, including completion of relevant forms • Follow-up often undertaken by peer-led outreach staff	Canada, New Zealand, Spain, UK (Scotland)^[c]^	Kenya, Myanmar, South Africa, Ukraine
Online access	• Can be provided by non-governmental organizations (NGOs) or the private sector • Online outreach workers provide instructions on how to obtain services at NGOs or affiliated clinics, including for those who wish to access PrEP privately • Access to anonymous PrEP assessment tools and/or online counselors • Online purchase of PrEP medication		Russian Federation, Ukraine

^a^PrEP services for people who inject drugs were also identified in Georgia, Guatemala, and North Macedonia. However, insufficient data were available for these countries to categorize service delivery models

^b^Countries were classified according to World Bank country income levels as of December 28, 2022: https://datahelpdesk.worldbank.org/knowledgebase/articles/906519-world-bank-country-and-lending-groups

^c^Due to the devolved system of the UK, PrEP service delivery models for people who inject drugs varied across England and Scotland (and none were identified in Wales and Northern Ireland)

## Discussion

Our study identified 27 countries where PrEP services were provided or planned for people who inject drugs, most of which were high-income countries; however, in many countries PrEP services were small scale and restricted to specific geographic areas or cities and data regarding coverage were limited. Service delivery approaches differed widely within and across countries. This included services provided in healthcare clinics and outside of clinic settings, services involving peers in outreach activities, and integration with other harm reduction services. Most of the identified PrEP services for people who inject drugs required an HIV or sexual health specialist doctor to prescribe PrEP. However, task sharing with nurses in clinics and community settings to lead PrEP services, including prescribing, has the potential to increase PrEP access, uptake and persistence and generate health system efficiencies [29] and has been implemented in South Africa. Along with appropriate training and support, nurse-led models could be an effective option for providing PrEP for people who inject drugs, including within broader HIV, tuberculosis and STI prevention, diagnosis and treatment services, and in services for HCV infection, particularly where nurses work with civil society organizations providing harm reduction services to people who inject drugs and other key populations and so tend to be familiar with the legal, socio-cultural, political, and health contexts for people who inject drugs.

A comprehensive and integrated approach to PrEP service delivery is needed to address multiple health and social needs of people who inject drugs. Although PrEP services for people who inject drugs identified in this study were most commonly provided in standalone, fixed healthcare settings, a range of community-based services providing PrEP outside of clinic settings and peer-led services were also identified. These community-based and peer-led models are likely to be most acceptable to people who inject drugs [15, 26, 30, 31], increasing access to and uptake of PrEP by people who inject drugs. However, there are challenges to implementing integrated PrEP services. While it is recommended to integrate PrEP and other HIV services into harm reduction programs, less than 1% of people who inject drugs live in countries with high coverage of both NSPs and OAMT (>200 needle/syringes distributed per person who injects drugs and >40 OAMT recipients per 100 people who use opioids) [3].

Dispensing PrEP through community pharmacies may increase accessibility for individuals and reduce costs [31]. In several countries, community pharmacies already dispense sterile injecting equipment and OAMT [32–34]. Pharmacies may also be able to work with community healthcare workers to support PrEP persistence. However, structural barriers such as criminalization, stigma, and discrimination limit access to pharmacy services for people who inject drugs [35] and in order for pharmacies to improve access to PrEP they need to provide services in a non-judgmental, non-stigmatizing way, and understand the structural impediments and diverse health and social needs of people who inject drugs.

People who inject drugs may also choose to access PrEP information and order or purchase PrEP drugs online, either due to a lack of availability through other venues, for convenience, or anonymity. Our study found this to be implemented in the Russian Federation and Ukraine. While use of online approaches to accessing health services is acceptable by some people who inject drugs [36], particularly young people [37], studies have highlighted barriers to access experienced by others who have lower levels of access to devices, technology, mobile data, and internet connections [38–40], especially older people who inject drugs and those who are homeless [41]. The COVID-19 pandemic further exacerbated these access barriers as many services moved to online health provision [42–45], reducing access for some. While online services may increase access to PrEP for some people who inject drugs, it is important that services offer linkages to other services as needed, including evidence-based harm reduction.

All PrEP services identified in this study focused on oral PrEP. In 2021, WHO recommended the dapivirine vaginal ring (DVR) as an additional HIV prevention option for cisgender women [46]. One qualitative study found that the DVR may be an acceptable option for women who inject drugs to reduce risks of HIV acquisition through sexual exposure in some regions but highlighted that, despite interest, it was either unavailable or women who inject drugs or women were unsure about their availability [15]. Long-acting injectable cabotegravir (CAB-LA) is another PrEP option recommended for HIV prevention by WHO [47]. WHO guidelines, however, note that none of the studies on CAB-LA have included people who inject drugs to-date. The efficacy and acceptability of CAB-LA in this population is therefore uncertain and implementation science involving offering oral PrEP and CAB-LA options for people who inject drugs is needed, recognizing that PrEP must always be a complement to evidence-based harm reduction services.

A limitation of this study is that the list of identified projects may not be exhaustive and countries where PrEP is available for people who inject drugs may have been missed, although a range of professional networks were contacted to identify PrEP services for people who inject drugs. Moreover, there was often scarce information on PrEP services. There were no or limited data on acceptability of and uptake of PrEP through different service delivery models across countries, so their effectiveness could not be evaluated, although integrated, community-based and peer-led services were likely most acceptable to people who inject drugs. In addition, both the self-identification and the identification by PrEP providers of an individual as being a person who injects drugs can be problematic. For instance, individuals may self-identify and present themselves to service providers as men who have sex with men first and as a person who injects drugs second or not at all. Therefore, our study likely missed PrEP services that included people who inject drugs but were not identified as such.

## Conclusions

Harm reduction services are key to reducing potential health risks associated injecting drug use and PrEP can be an additional HIV prevention tool for people who inject drugs, particularly for those with sexual behaviors that may also increase their HIV risk. This study indicates limited availability of PrEP services for people who inject drugs globally. Services outside of health clinic settings and peer-led services are likely most acceptable to people who inject drugs, with communities of people who inject drugs actively involved in designing and delivering PrEP services. Comprehensive harm reduction programs that include the provision of sterile injecting equipment, OAMT and community-based naloxone as well as condoms and other prevention interventions are critical to reducing the risk of HIV acquisition and to addressing other health needs of people who inject drugs, particularly HCV and STIs, which PrEP does not prevent. Renewed effort, and investment, is needed globally to ensure availability, accessibility, and acceptability of harm reduction services that can serve as a strong foundation to the introduction of additional services including PrEP. Harm reduction advocacy organizations have expressed concerns about making antiretroviral drugs available for prevention when there are insufficient drugs available for treatment for people who inject drugs who are living with HIV [15], indicating that ongoing advocacy and consultation with people who inject drugs is crucial for planning and promoting PrEP services for people who inject drugs. Integration of PrEP services may provide an opportunity to increase investment in and strengthen delivery of a comprehensive harm reduction program. Addressing structural barriers to harm reduction service availability, accessibility, and acceptability, especially the decriminalization of people who inject drugs, remains critical.

## Supplementary Information

**Additional file 1**. Details on methods.

## Data Availability

The dataset supporting the conclusions of this article is available on request from the corresponding author (R Schaefer: schaeferr@who.int).

## Abbreviations

HCV: Hepatitis C virus
NSPs: Needle and syringe programs
OAMT: Opioid antagonist maintenance therapy
PrEP: Pre-exposure prophylaxis
STI: Sexually transmitted infection
TDF: Tenofovir disoproxil fumarate
WHO: World Health Organization
