# Supplementary material for: Pre-exposure prophylaxis (PrEP) for HIV prevention among people who inject drugs: a global mapping of service delivery
Source: Harm Reduct J. 2023 Feb 13;20:16. doi: 10.1186/s12954-023-00729-6 (PMC9924874; doi:10.1186/s12954-023-00729-6)
Supplement: Supplementary file 1 — Additional file 1. Details on methods. [file 12954_2023_729_MOESM1_ESM.pdf]

# Pre-exposure prophylaxis (PrEP) for HIV prevention among people who inject drugs: A global mapping of service delivery

## *Additional file 1: Details on methods*

Graham SHAW, Robin SCHAEFER, Heather-Marie A SCHMIDT, Annie MADDEN, Judy CHANG, Antons MOZALEVSKIS, Busisiwe MSIMANGA-RADEBE, Nabeel MANGADAN KONATH, Annette VERSTER, Rachel BAGGALEY, Michelle RODOLPH, Virginia MACDONALD

In this additional file, further details on the methods for data collection are outlined.

***Outreach to professional networks and organisations***
Using the professional networks of a range of individuals and organisations working in the sectors of HIV/AIDS, sexual and reproductive health and rights (SRHR), harm reduction and human rights around the world, information was collated as to the programmes and organisations delivering PrEP services for PWID. Once identified, such PrEP service providers were contacted directly and asked to reply to a standard set of questions (see below), the responses to which were used to map key components of the respective service.

***Outreach to online groups***
Requests for information were issued to various online groups including the Stimulant Expert Group, the Opioid Safety and Naloxone Network and the PrEP Fact group as well as through various regional Facebook groups including those of the European Network of People who Use Drugs (EuroNPUD) and the AfricaNPUD. Contact was also made by email with other networks of people who use drugs including the Asian Network of People who Use Drugs (ANPUD) and the Southern Africa Network of People who Use Drugs (SANPUD).

***Website search***
A manual search was undertaken between October and December 2021 of websites of key organisations involved in HIV prevention and PrEP-related studies or services for people who inject drugs including, for example, the Global Advocacy for HIV prevention (AVAC) and its associated PrEP Watch and the October 2021 version of the Global PrEP Tracker[1], as well as a review of PrEP MAP[2] for the Asia-Pacific region.

***Standard set of questions for data collection***
The following standard set of questions were used to collect data from the literature and other sources as part of the mapping of PrEP services for people who inject drugs:

1. How many fixed site and mobile facilities (or through other mechanisms) does your organisation use to provide PrEP to people who inject drugs?
2. Who is authorised to assess the eligibility of an individual for enrolment onto PrEP?
3. Who prescribes the PrEP medication (e.g. doctor, nurse, other)?
4. Who dispenses the PrEP medication? And where (community pharmacy, hospital, doctor's clinic, etc.)?
5. What PrEP guidelines do you use?
6. Who pays the cost of the PrEP medication, laboratory work, etc.? If it is free to the client, who ultimately pays the costs incurred by your organisation?
7. Is PrEP part of an integrated package of harm reduction services, or is it a stand-alone service?
8. How do you monitor the individual who is prescribed PrEP?
9. On average, how many people who inject, or otherwise use, drugs receive PrEP each month from your organisation (newly enrolled and those already enrolled)?

[1] AVAC. Global PrEP Use Landscape as of October 2021. https://www.prepwatch.org/wp-content/uploads/2021/10/2021_Q3_PW_GlobalTracker.xlsx (accessed 25 January 2022).
[2] APCOM. PrEPMap Asia-Pacific. Bangkok; APCOM https://www.prepmap.org/ (accessed 25 January 2022).

***Data parameters***

Collected data on PrEP service delivery were organised as follows:

| | | | | | |
|---|---|---|---|---|---|
| **Location(s) of service provision** (town/city; province; country) | | | | | |
| **Type of programme:** | | | | | |
| a) research | b) pilot / demonstration | c) national | d) other | | |
| **Duration of PrEP provision:** | | | | | |
| a) time-limited | b) ongoing | c) planned for future implementation | | | |
| **Number of clients in receipt of PrEP services (and timeframe)** | | | | | |
| **Population group(s) targeted by the PrEP service:** | | | | | |
| a) people who inject drugs | b) people who use drugs | c) other (specify) | | | |
| **PrEP service enrolment model:** | | | | | |
| a) clinic-based | b) community-based | c) HIV specialist | d) primary care | e) peer / online-based | f) other (specify) |
| **PrEP prescribing approach:** | | | | | |
| a) clinical HIV/STI doctor | b) other clinical doctor | c) nurse | d) pharmacist | e) other (specify) | |
| **PrEP dispensing approach:** | | | | | |
| a) HIV/STI/infectious disease clinic | b) pharmacy | c) community setting | d) other (specify) | | |
| **PrEP funding:** | | | | | |
| a) national programme with co-payments | b) national programme without co-payments | c) private prescription and online purchase | d) donor | e) other (specify) | |
| **Guidelines used:** | | | | | |
| a) in accordance with WHO or CDC recommendations and good practices | b) other (specify) | | | | |
